# Model-Based Evaluation of Highly and Low Pathogenic Avian Influenza Dynamics in Wild Birds

**DOI:** 10.1371/journal.pone.0010997

**Published:** 2010-06-23

**Authors:** Viviane Hénaux, Michael D. Samuel, Christine M. Bunck

**Affiliations:** 1 Department of Forest and Wildlife Ecology, University of Wisconsin, Madison, Wisconsin, United States of America; 2 Wisconsin Cooperative Wildlife Research Unit, United States Geological Survey, Madison, Wisconsin, United States of America; 3 National Wildlife Health Center, United States Geological Survey, Madison, Wisconsin, United States of America; University of Leeds, United Kingdom

## Abstract

There is growing interest in avian influenza (AI) epidemiology to predict disease risk in wild and domestic birds, and prevent transmission to humans. However, understanding the epidemic dynamics of highly pathogenic (HPAI) viruses remains challenging because they have rarely been detected in wild birds. We used modeling to integrate available scientific information from laboratory and field studies, evaluate AI dynamics in individual hosts and waterfowl populations, and identify key areas for future research. We developed a Susceptible-Exposed-Infectious-Recovered (SEIR) model and used published laboratory challenge studies to estimate epidemiological parameters (rate of infection, latency period, recovery and mortality rates), considering the importance of age classes, and virus pathogenicity. Infectious contact leads to infection and virus shedding within 1–2 days, followed by relatively slower period for recovery or mortality. We found a shorter infectious period for HPAI than low pathogenic (LP) AI, which may explain that HPAI has been much harder to detect than LPAI during surveillance programs. Our model predicted a rapid LPAI epidemic curve, with a median duration of infection of 50–60 days and no fatalities. In contrast, HPAI dynamics had lower prevalence and higher mortality, especially in young birds. Based on field data from LPAI studies, our model suggests to increase surveillance for HPAI in post-breeding areas, because the presence of immunologically naïve young birds is predicted to cause higher HPAI prevalence and bird losses during this season. Our results indicate a better understanding of the transmission, infection, and immunity-related processes is required to refine predictions of AI risk and spread, improve surveillance for HPAI in wild birds, and develop disease control strategies to reduce potential transmission to domestic birds and/or humans.

## Introduction

Avian influenza (AI) became a significant human and domestic animal health issue in 1996 when highly pathogenic (HP) H5N1 virus was isolated from domestic geese in southern China. Since 2002, HPAI H5N1 strains have emerged in both wild and domestic birds and spread throughout the Old World [1]–[2]. Concern for interspecies transmission and adaptation of AI viruses to mammalian hosts has renewed interest in the epidemiology of AI in wild and domestic birds to predict disease risk and spread, and prevent further transmission to human and domestic populations.

Historically, HPAI viruses in domestic poultry, and subsequently humans, likely arose by the introduction and mutation of low pathogenic (LP) AI strains from wild birds. Extensive field surveys reported the continuous worldwide circulation of LPAI in wild birds, primarily Anseriformes and Charadriiformes, which are considered the natural reservoir of all 16 HA and 9 NA subtypes of influenza A viruses [3]–[5]. Prevalence and distribution of LPAI virus subtypes markedly differ among species, years, and places. Within bird migratory flyways, a seasonal decline in LPAI prevalence has been typical, with higher prevalence in naïve young birds [6]–[9]. The components underlying spatial and temporal heterogeneity in AI prevalence are still unclear. Epidemic dynamics may be influenced by ecological factors that influence habitat use by waterfowl species, species differences in susceptibility, age- and species-related differences in virus shedding, environmental conditions that influence virus persistence in wetlands [10], and immunity (or cross-immunity) from a previous exposure to influenza viruses [11].

There is currently little scientific information about the basic epidemiology of AI in wild and domestic birds, the role of the environment as a reservoir for AI, the mechanisms underlying AI immunity, and the potential role of wild birds as carriers of HPAI viruses. The impact of HPAI in wild birds is species-specific and some migrating species shed large amounts of virus without exhibiting clinical disease [12]–[17], suggesting that they could serve as long-distance carriers. Assessing the temporal and spatial dynamics of AI viruses, the potential impact of HPAI on wild birds, and the risk of HPAI transmission and spread by migratory birds is challenging because HPAI research is restricted to high security laboratories and natural epidemics are rare; typically discovered after epidemiological dynamics have occurred and/or affected wild birds have migrated.

Understanding AI dynamics in wild bird populations can be facilitated by using models that evaluate and integrate diverse scientific information from laboratory and field studies. In addition, models of disease systems can help to assess the potential effectiveness of alternative management strategies [18], identify critical gaps in our knowledge, and determine relative importance of different parts of the system [19]. We developed an epidemiological SEIR model (Susceptible, Exposed, Infected, Recovered) that describes the dynamics of AI infection in individual birds. AI viruses are transmitted by bird-to-bird contact or through the environment. After infectious contact, a susceptible (S) bird becomes exposed (E) or infected. During the latent period, AI virus develops within the host until the host becomes infectious (I) by shedding virus. LPAI viruses replicate preferentially in the gastrointestinal tract and are excreted at high levels in the feces. In contrast recent HPAI viruses, and in particular H5N1 viruses isolated since 2002, replicate primarily in the upper respiratory tract of wild ducks [15]. Ultimately, the host will recover (R) or die (D) depending on the interaction between its immune system and the virus.

Parameters for the SEIR model, including rate of infection, latent period, recovery and mortality rates are estimated from analyses of published laboratory infection trials for different Anseriform species and AI viruses. In our assessment, we evaluate age-related differences in disease parameters to compare AI dynamics in post-breeding populations composed of hatch-year (<1.5 months) and adult birds, and wintering populations (adult birds only). We also consider the effect of virus pathogenicity on epidemiological parameters. We extended this model to wild bird populations by estimating the rate of infectious contact with virus (bird-to-bird or environmental contact) using prevalence data from waterfowl surveys. We use the SEIR model to evaluate the sensitivity of parameter estimates on dynamics during AI outbreaks, compare LPAI and HPAI outbreak predictions to field observations, and consider the potential for bird mortality and disease spread depending on the age-composition of the population.

## Results

### SEIR transition rates and times

Exponential rates of transition from susceptible (δ) or exposed (σ) to the infectious state were estimated from published laboratory challenge data (Table 1; see Figure S1 for exponential curve fit to the data). Susceptible birds exposed by contact to LPAI-inoculated birds became infectious at a slower rate (median time  = 1.50 days) compared with HPAI-inoculated birds (0.76 day). LPAI-inoculated birds also have a longer latent period (1.21 days) than HPAI-inoculated birds (0.28 day). Subtracting the latent time (i.e. E→I) from the time for susceptible individuals to become infectious (S→I), we estimated the median time required for susceptible birds to become exposed/infected (S→E), which was slightly longer for HP than LP viruses (0.48 vs. 0.28 day, respectively). We believe calculating S→E as a difference between other parameters combined with the scarcity of LPAI data produced an imprecise estimate for the LPAI infection rate, with a large SD. In HPAI laboratory challenges, the median time to become infectious was longer in young than adult birds for both contact (0.91 vs. 0.23 day, respectively) and inoculated birds (0.62 vs. 0.28 day, respectively). Similarly, in LPAI experiments, it took longer for susceptible young birds to become infectious compared to adults (1.83 vs. 0.89 days, respectively). Low sample size prevented the estimation of infection rates for young and adults with LPAI and for adults with HPAI. Overall, the infection process (S→I) was rapid, about a day with HPAI and two days for LPAI because of a longer latent period (E→I).

**Table 1:** Estimated transition rates and median time periods between epidemiological states for AI in waterfowl.

		HP			LP		
Parameter		Adult	Young	Both ages	Adult	Young	Both ages
δ (S→I)	rate±SD	2.98±1.98	0.76±0.07	0.92±0.10	0.78±0.41	0.38±0.15	0.46±0.11
	median time (day)	0.23	0.91	0.76	0.89	1.83	1.50
		*n* = 6	*n* = 69	*n* = 75	*n* = 3	*n* = 3	*n* = 6
σ (E→I)	rate±SD	2.47±0.40	1.12±0.15	2.47±0.37	0.54±0.13	†	0.57±0.13
	median time (day)	0.28	0.62	0.28	1.28		1.21
		*n* = 28	*n* = 78	*n* = 106	*n* = 7	*n* = 1	*n* = 8
τ (1) (S→E)	rate±SD	n/a	2.39±0.99	1.46±0.29	n/a	n/a	2.44±3.96
	median time (day)		0.29	0.48			0.28
γ (I→R)	scale±SD	4.62±1.17	5.32±1.07		9.81±1.30	11.28±1.19	
	shape	2.46	2.46		2.46	2.46	
	median time (day)	4.62	5.32		9.81	11.28	
		*n* = 23	*n* = 96		*n* = 8	*n* = 5	
*d* (I→D)	scale±SD	17.90±1.29	5.16±1.09		n/a	n/a	
	shape	2.26	2.26				
	median time (day)	17.90	5.16				
		*n* = 23	*n* = 96				

Rates estimated from published laboratory challenge trials, see methods for details.

*n*, number of laboratory challenges; n/a, not applicable; †, non-convergence of the model.

(1) By subtracting the latent period (1/σ) from the period for susceptible birds to become infectious (1/δ), we estimated the time for susceptible to become exposed (1/τ) and the infection rate τ.

All birds recovered from LPAI infection, so the mortality rate was nil in LPAI-infected birds. Among parametric survival models, the loglogistic model provided the best fit (lowest AIC) to the HPAI time to mortality data (Table S1 and Figure S2). The median time to death was significantly shorter (*P*<0.001) in young than adult HPAI-infected birds (Table 1). The loglogistic was also the best model for the time to recovery data (Table S1 and Figure S2). The median recovery period was markedly shorter in HPAI- than LPAI-infected birds (*P*<0.001). Recovery tended to be shorter in adult birds than young (*P* = 0.13, Table 1); however, there was substantial overlap in the estimated confidence intervals between adults and young for both HPAI and LPAI. For infectious individuals, mortality and recovery are competing outcomes which simultaneously influence the length of the infectious period. Although median recovery times from HPAI are similar between young and adult waterfowl (4.62 vs. 5.32 days), the median mortality period was four times longer in adults than young (17.90 vs. 5.16 days). As a result, the median infectious period is slightly longer in adult (4 days) than young HPAI-infected birds (3 days), and more fatalities are expected in young than adult birds. Moreover, the longer recovery period for LPAI results in a longer median infectious and shedding period (about 10 days) than for HPAI (3.5 days).

### Sensitivity and AI dynamics

Sensitivity analysis using Latin hypercube sampling indicated that peak prevalence (maximum proportion of infectious birds) was most sensitive to the rate of infectious contact θ and rate of infection τ (Table S2). Higher infectious contact or infection rates produced more rapid epidemics with higher peak prevalence. However, the amount of population mortality caused during AI epidemics was most sensitive to recovery and mortality rates. Lower recovery rates or higher mortality rates for individual birds produced an increase of the number of dead birds.

**Figure 1:**
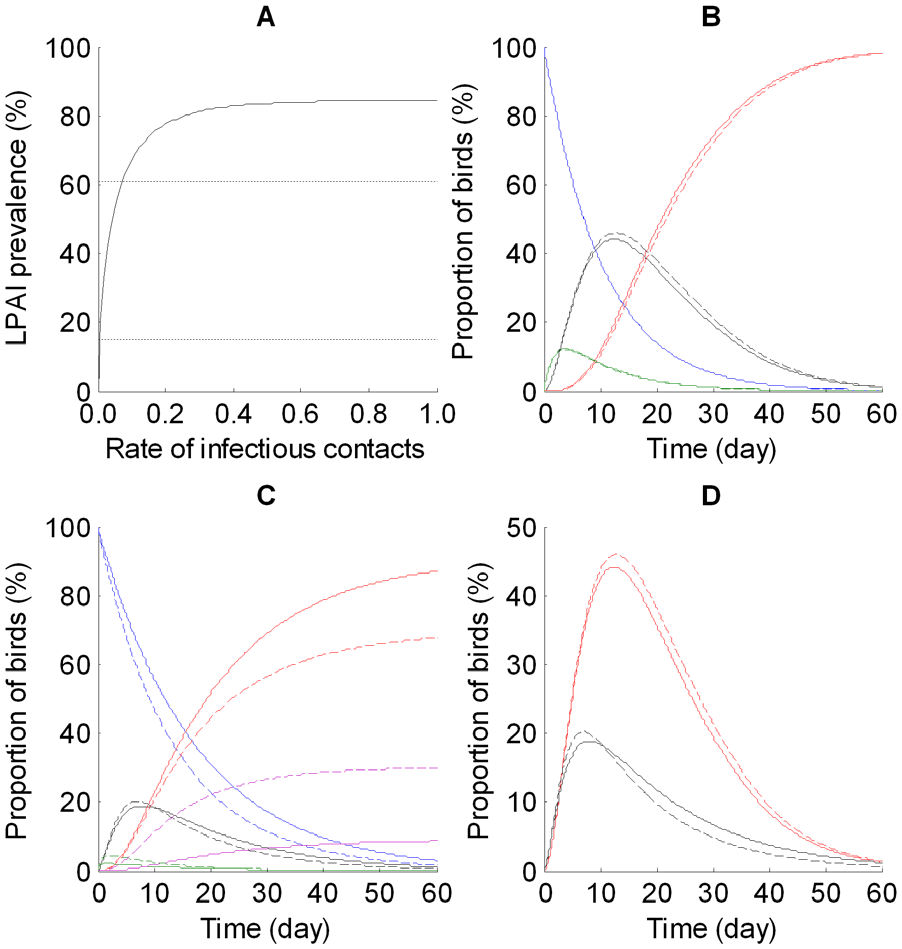
Predicted prevalence and disease dynamics in wild birds. A: LPAI peak prevalence for hatch-year waterfowl and estimated rate of infectious contact θ; θ values between 0.01 and 0.08 corresponded with LPAI prevalence observed in young waterfowl at post-breeding areas (15–61%, delimited by dotted lines). B and C: Predicted dynamics during LPAI and HPAI outbreaks, respectively, for Susceptible (blue), Exposed (green), Infectious (black), Recovered (red), and Dead (purple) birds in post-breeding (dashed lines) and wintering populations (solid lines). Graphs based on mean epidemiological parameters (Table 1) and an infectious contact rate (θ) of 0.04. D. Comparison of prevalence dynamics during LPAI (red) and HPAI (black) epidemics in post-breeding (dashed lines) and wintering populations (solid lines).

We investigated LPAI and HPAI dynamics in post-breeding and wintering waterfowl populations, using mean estimates for epidemiological parameters (Table 1). Because recovery and mortality rates from the loglogistic model depend on the time since infection, we implemented our model using 30 daily sub-stages of the infectious period, each with a corresponding loglogistic hazard rate, to estimate rates of recovery (or mortality) at each day since infection. LPAI prevalence predicted by the model was similar to field prevalence estimates reported in young wild ducks (15–61%) for θ values ranging from 0.01 to 0.08 (Figure 1A). In our model, θ represents the daily probability that any susceptible wild bird in the population will have an infectious contact. Peak prevalence when all birds were exposed on day one (θ = 1) reached 85%. We used a mean rate of infectious contact of 0.04 to further investigate disease dynamics in waterfowl populations. Our model predicted rapid LPAI epidemic curves with peak prevalence of about 45% 13 days after the initial infection (Figure 1B). There was no difference in predicted seasonal LPAI dynamics because of the lack of information on age-specific infection rates (τ and σ; Table 1). The median duration of a LPAI epizootic was about 50–60 days with no fatalities. HPAI dynamics, in contrast to LPAI, were characterized by a shorter epidemic curve, lower peak prevalence, and high bird losses particularly in the post-breeding population (Figure 1C). The post-breeding population had peak prevalence of 20% at day 7 and 30% of individuals were dead 60 days after the onset of the HPAI outbreak. In the wintering population, HPAI prevalence reached 19% at day 8 but the proportion of susceptible birds that died during the epidemic was lower (about 9%) than at post-breeding areas (Figure 1D). The median duration of HPAI epizootics was about 40–50 days.

## Discussion

Based on a comprehensive analysis of published data on HPAI and LPAI for waterfowl, we estimated transition rates between disease states including the rates of infection, disease progression within the host (latent period), host mortality and recovery. We implemented disease transition rates using an epidemiological model that provides the foundation for understanding and predicting the dynamics of AI outbreaks in bird populations. In individual birds, contact with AI-infectious birds and/or a contaminated environment leads to rapid progression of infection and virus shedding within 1–2 days, followed by relatively slower periods of recovery or mortality. We found a higher mortality rate in young than adult HPAI-infected waterfowl, but similar recovery rates. Age-related difference in the outcome of infection has previously been observed in ducks infected with AI [20]–[21] or other viruses [22]. Hypothesized mechanisms for age-related differences include maturation of the immune system or links between host cell maturation and the capacity for virus replication [22].

To predict disease dynamics in wild birds, we approximated the rate of infectious contact θ from LPAI prevalence data in wild populations of young (naïve) ducks. Our model facilitates a comparison of AI dynamics and showed substantial differences in the dynamics of LPAI and HPAI infections, with shorter HPAI epidemics, lower prevalence of infectious birds, and higher mortality than LPAI epidemics. Because we used similar rates of infectious contact θ, the non-mortality differences likely reflect a longer latent period (E→I) and longer recovery period (I→R) for LPAI (Table 1). Although we assumed infectious contact rates were constant over time and similar in both LP and HPAI outbreaks, the rate of infectious contact likely depends on the relative importance of bird-to-bird and environmental transmission, number of infected birds, abundance of virus in wetlands, and inherent characteristics of the host and virus. LPAI viruses multiply primarily within the digestive tract, are shed into the environment where they persist for an extended period of time [10], and become a critical reservoir for sustaining LPAI virus in waterfowl populations [23]. In contrast, HPAI viruses are shed via both oral and cloacal routes (due to replication in the respiratory and digestive tracts), but have rarely been isolated from the environment [24]. Consequently, the role of environmental transmission in HPAI epidemics remains unknown. Although we found a longer recovery period (longer infectious period) for LPAI than HPAI, we speculate that oral and cloacal shedding of HPAI viruses may help increase transmission in spite of the relatively short infectious period. Given the significant effect of θ on epidemic dynamics (Table S2), determining the relative importance of alternative transmission routes, rates of infectious contacts, and factors that influence contact rates will enhance our understanding of AI dynamics in wild bird populations.

We found few differences in predicted epidemic dynamics based on time of year (i.e. mix of adult and young waterfowl in the population; Fig. 1B and C). Although the lack of age-specific challenge trails for LPAI prevents a reliable evaluation of seasonal differences in susceptibility to AI viruses, we found age-related differences in disease processes for HPAI, indicating faster disease progression (S→I and E→I) in adult than young birds. Our sensitivity analysis also demonstrated that the rate of infection (S→E) plays a key role in AI epidemic dynamics. We acknowledge that uncertainty in LPAI infection rate may have affected our sensitivity analysis; however, we found infection rate was also a key parameter affecting peak prevalence in HPAI dynamics (Table S2). Additional laboratory challenges clarifying age-related differences in the LPAI infection processes, the source of virus exposure (by fecal/oral transmission or environmental transmission), and the level of exposure (with dose-response experiments) would expand our understanding of infection rates under various conditions.

Because little is known about AI immunity in wild birds our model assumed the entire population is susceptible to infection, and that recovery confers immunity for the duration of an epidemic (>50–60 days). It has been observed that Pekin ducks (*Anas platyrhinchos*) have sufficient immunity to clear a secondary LPAI-infection without shedding virus for at least 12 weeks after initial infection with the same LPAI subtype [11]. It has also been demonstrated that birds with preexisting LPAI antibodies have a lower probability of developing HPAI infection, but may shed HP virus [16], [25]–[28]. The predicted effect of immunity (or cross-immunity) is a decline in the proportion of susceptible birds and an increase in the proportion of recovered and resistant birds as LPAI viruses spread in the population during fall migration. However, the length of these effects also depends on the length of immunity (e.g. 3 months vs. one year) and extent of cross-immunity among AI viruses. The consequence is a decline in epidemic curve by decreasing the proportion of infectious birds (Figure 1A). A similar conclusion was illustrated in a previous simulation model of annual LPAI dynamics in dabbling ducks [29]. An improved understanding of the processes underlying immunity and cross-immunity among LP and HPAI viruses is important in assessing the susceptibility of wild bird populations to HPAI viruses, predicting when epidemics are likely to occur, and developing effective surveillance programs for HPAI.

Extensive surveillance programs for AI viruses have reported the presence of LPAI asymptomatic carrier birds all around the world (at various levels of prevalence depending on season, bird species, and virus strain). On the other hand, HPAI viruses have been detected in only a few healthy wild birds [30], and in most HPAI outbreaks, only a few dead individuals have been found. In a general sense, these observations agree with our model predictions of short epidemics for HPAI and much higher prevalence and longer duration of infection in birds with LPAI. Using the infectious contact rate for LPAI to predict HPAI dynamics, our model predicts a low epidemic curve on wintering areas, with negligible or scattered mortalities, which will likely be difficult to detect. In addition, we suspect that HPAI outbreaks may be even less conspicuous on wintering grounds because a large proportion of waterfowl would likely have immunity from previous LPAI infections. In contrast, our model predicts a higher HPAI prevalence (and mortality) in post-breeding areas. Because surveillance programs have primarily relied on swabs to detect AI in wild birds (e.g. [8], [31]–[32]), it is not surprising that HPAI, with a shorter length of infection than LPAI, has been much harder to detect. Our results suggest that serological surveys to determine circulation of AI viruses in avian populations [33] may be much more effective because AI antibodies appears to last considerably longer than infection. Overall, our model suggests intensifying AI surveillance in post-breeding areas, which have a large number of immunologically naïve birds, to increase the probability of detecting HP viruses, given the expected higher prevalence and fatalities in these areas.

## Methods

### Epidemiological model

We mathematically modeled AI dynamics for a closed biological system with no changes in bird demographics (no immigration, emigration, or natural mortality), where all of the initial population is susceptible, and birds that recover have long-term (> length of the epidemic curve) immunity. We used ordinary differential equations to model changes in the number of birds in each SEIR state with time *t*: 
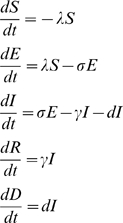



where λ is the force of infection, σ is the rate that latent birds become infectious, γ is the recovery rate (i.e. the instantaneous probability of recovering, conditional on survival to that time), and *d* is the disease-related mortality rate. The force of infection λ, the rate at which susceptible birds transition to the infected state (E), is the product of the rate of infectious contact θ (by bird-to-bird contact and environmental route) and the rate τ at which susceptible birds that contact virus become infected (E). We implemented the model using a daily time step (*t* = 1 day).

### Laboratory challenge trials

AI laboratory challenge data published over the three last decades were used to estimate model parameters [11], [13]–[17], [21], [25], [34]–[46]. We analyzed experiments where birds were “naturally” inoculated (i.e. via the nares or throat) and excluded data from intramuscular and intravenous challenges. We considered inoculated birds as exposed (latent) individuals, assuming that virus inoculation represented infectious exposure. In some experiments, susceptible birds were housed in direct contact with inoculated birds. We considered birds to be infectious when they began shedding virus (detected by virus isolation in oral or cloacal samples). Recovered birds were individuals which no longer shed virus. We only considered experiments where the course of the disease (i.e. disease state) was measured at least once within four days post-challenge.

We evaluated disease dynamics in waterfowl (i.e. Anseriformes) infected with viruses isolated from birds or humans. We considered two age classes, <1.5 month (young) and ≥1.5 month (adult), to evaluate age-related differences in disease parameters and to compare AI dynamics in post-breeding and wintering populations. We also considered viruses as HP or LP, based on their ability to cause disease in chickens [47].

### SEIR transition rates

We estimated the rate σ at which exposed birds become infectious from the cumulative proportion of infectious birds through time (number of infectious birds/number of birds in the experiment) from challenge trials with inoculated birds. Similarly, we estimated the rate δ at which susceptible birds become infectious from challenge trials where susceptible birds had contact with infected birds. We estimated σ and δ using nonlinear mixed-effects models in R 2.1.1 [48]–[49] assuming a constant transition rate, which produces an exponential distribution of the proportion infected:



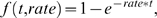



where *rate* denotes the transition rates σ or δ. From laboratory studies we found ≥97% of the contact birds become infected. We estimated the period for susceptible birds to become exposed (transition rate τ) by subtracting the latent period (transition E to I) from the period for susceptible birds to become infectious (S to I).

Because recovery and death are competiting events, we used survival analyses to estimate recovery γ and disease-related mortality *d* rates for infectious birds. The time to recovery or mortality was right-censored when the individual was still infectious at the end of the experiment or was removed (in an infectious state) before the end of the experiment. We evaluated several parametric hazard models including the Exponential, Gaussian, Logistic, Loglogistic, and Weibull [50] using Akaike Information Criterion (AIC [51]). Goodness-of-fit for the selected model was assessed using the Grønnesby and Borgan [52] test, which compares the number of observed events with those expected from the model, within four risk-score groups for the recovery data (206 events) and two risk-score groups for the mortality data (72 events). We incorporated covariates in the hazard rate using the model scale parameter:



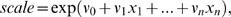



with ν_0_ the coefficient of the intercept, and ν*_n_* the coefficient of the covariate *x_n_* [53].

### AI dynamics and sensitivity

We tested the potential importance of epidemiological parameters on model predictions using Latin Hypercube Sampling [54]. This stratified Monte Carlo sampling procedure identifies the key parameters which affect model outputs. In this method, the assumed probability distribution of each model parameter is divided into *N* equal probability intervals and one random value is selected from each interval. The *N* values obtained for each parameter are paired randomly with the *N* values from all other parameters. In our sensitivity analysis, we used *N* = 30, a uniform distribution *U*(0,1) for the rate of infectious contact θ, and Gaussian distributions *N*(mean, SD^2^) for the infection rate τ, the transition rate σ, and scale parameters of the recovery rate γ, and disease mortality rate *d*. Mean parameter estimates and SD are given in Table 1. Given the large SD for the LPAI infection rate, we used a uniform distribution *U*(0, 10) for this parameter. We quantified parameter sensitivity on model predictions for the number of infectious birds at peak prevalence (peak of the epidemic curve) and cumulative number of dead birds during the epidemic. We measured sensitivity using the semipartial correlation coefficient, SPC, which measures the linear correlation between a model parameter and output, corrected for other correlated parameters [55]. Sensitivity was based on 100 model runs for 60 days.

In laboratory challenges, susceptible (contact) birds were exposed to AI virus by close contact with infectious individuals and a contaminated environment. Therefore, the rate of infectious contact θ is assumed to be 1. To model AI dynamics in wild bird populations with a realistic θ value, we estimated the range of θ which predicts LPAI prevalence reported in young wild ducks (all assumed susceptible) at post breeding areas (15–61% [56]–[59]). We assumed that field estimates correspond to peak prevalence. We used the mean value for θ in our model to investigate LPAI and HPAI dynamics in waterfowl populations of 10000 birds, composed of 50% adult and 50% young birds (approximate post-breeding population composition; [60 p. 230]) or of adult birds only (wintering population). Scarcity of challenge trials prevented us from estimating age-related infection rates (LPAI and HPAI) and latent period (LPAI); therefore, we used mean estimates from both age classes (Table 1) in model projections and sensitivity analyses.

## Supporting Information

Figure S1 Estimated LP and HPAI infection rates. Proportion of young (black) and adult (grey) birds which become infectious by exposure to infected birds or a contaminated environment (top), or after inoculation with AI (bottom). Lines represent exponential curves for mean infection rates provided in Table 1. Marker size is proportional to the number of experimental challenges (1 to 70). Note different time scales among graphs.
(0.23 MB TIF)

Figure S2 Cumulative recovery and mortality probabilities after infection. Lines with markers are cumulative probabilities of young (black) and adult (grey) birds from laboratory challenges. Corresponding lines without markers are the predicted cumulative probabilities for the loglogistic model based on mean recovery and mortality rates provided in Table 1.
(5.76 MB TIF)

Table S1 Comparison of alternative parametric models for time to recovery and time to death. Akaike Information Criterion (AIC) values and number of model parameters (K) for alternative models of time to recovery and time to death from LP and HPAI laboratory challenge trials. Recovery data was modeled using an additive effect of age and virus pathogenicity (LP vs. HP). Time to death models only included HPAI-infected birds because LPAI infection did not cause mortality; these models also included the effect of age.
(0.04 MB DOC)

Table S2 Sensitivity of epidemiological parameters for peak prevalence and proportion of the population dying from AI. The model describes disease dynamics in a population of 10000 individuals during 60 days. We used Latin Hypercube Sampling (N = 30, 100 runs) and a semi partial correlation coefficient (SPC) to measure the relative influence of model parameters. See text for additional information.
(0.09 MB DOC)
