# Supplementary figures and images for: Model-Based Evaluation of Highly and Low Pathogenic Avian Influenza Dynamics in Wild Birds
Source: PLoS One. 2010 Jun 23;5(6):e10997. doi: 10.1371/journal.pone.0010997 (PMC2890401; doi:10.1371/journal.pone.0010997)

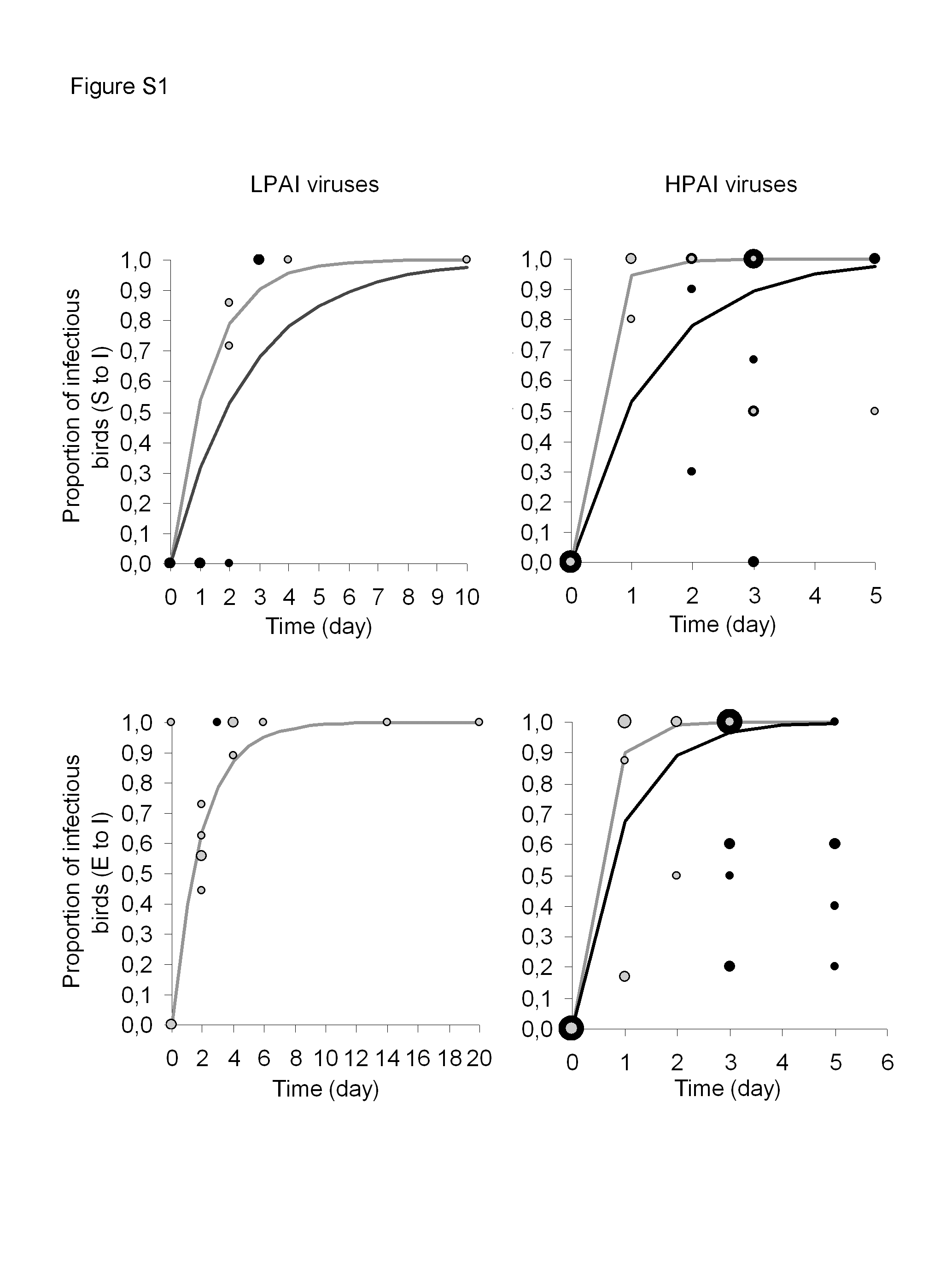

Supplement: Figure S1 — Estimated LP and HPAI infection rates. Proportion of young (black) and adult (grey) birds which become infectious by exposure to infected birds or a contaminated environment (top), or after inoculation with AI (bottom). Lines represent exponential curves for mean infection rates provided in Table 1. Marker size is proportional to the number of experimental challenges (1 to 70). Note different time scales among graphs. (0.23 MB TIF) [file pone.0010997.s001.tif]

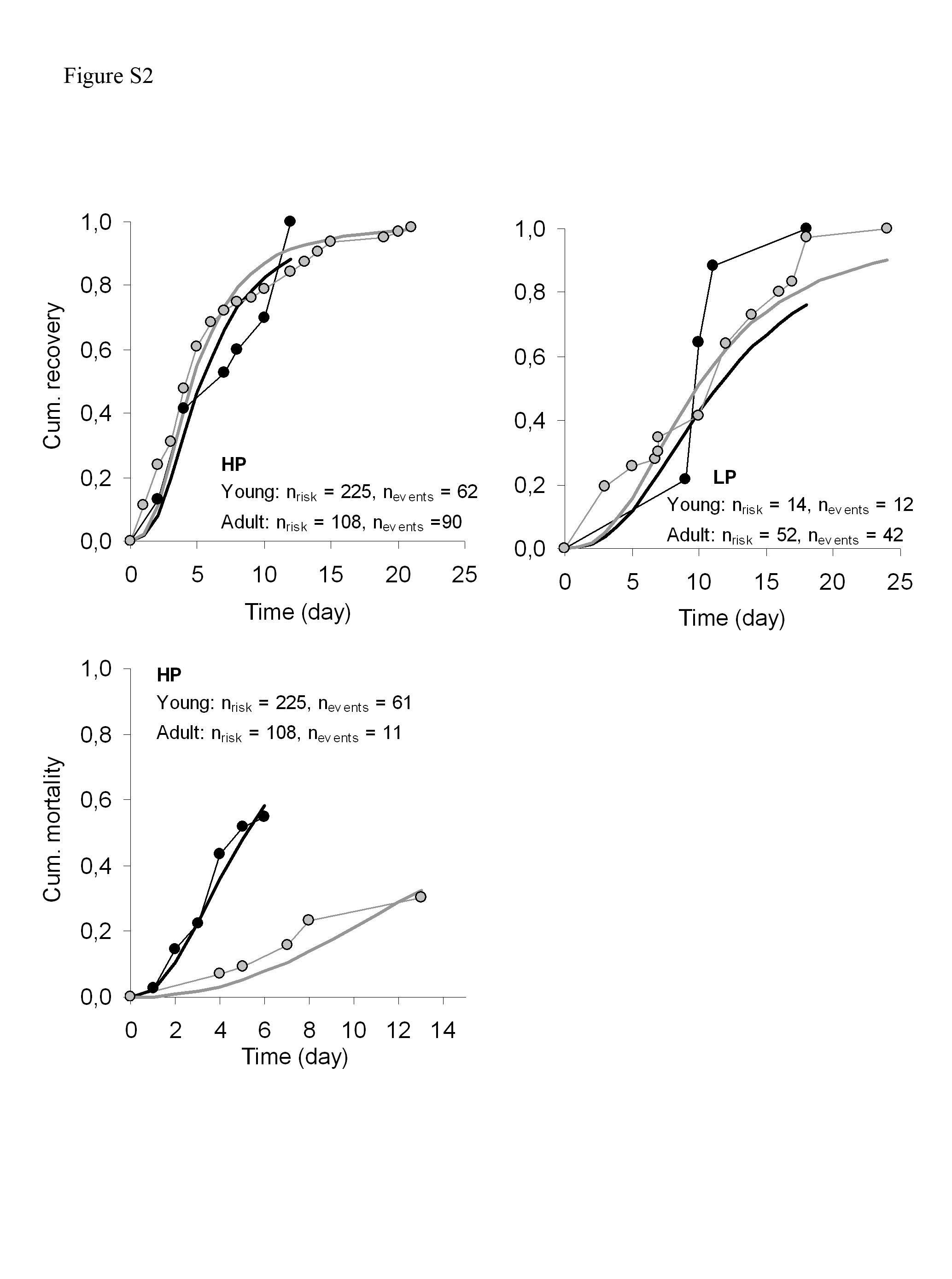

Supplement: Figure S2 — Cumulative recovery and mortality probabilities after infection. Lines with markers are cumulative probabilities of young (black) and adult (grey) birds from laboratory challenges. Corresponding lines without markers are the predicted cumulative probabilities for the loglogistic model based on mean recovery and mortality rates provided in Table 1. (5.76 MB TIF) [file pone.0010997.s002.tif]
