# Supplementary material for: Model-Based Evaluation of Highly and Low Pathogenic Avian Influenza Dynamics in Wild Birds
Source: PLoS One. 2010 Jun 23;5(6):e10997. doi: 10.1371/journal.pone.0010997 (PMC2890401; doi:10.1371/journal.pone.0010997)
Supplement: Table S1 — Comparison of alternative parametric models for time to recovery and time to death. Akaike Information Criterion (AIC) values and number of model parameters (K) for alternative models of time to recovery and time to death from LP and HPAI laboratory challenge trials. Recovery data was modeled using an additive effect of age and virus pathogenicity (LP vs. HP). Time to death models only included HPAI-infected birds because LPAI infection did not cause mortality; these models also included the effect of age. (0.04 MB DOC) [file pone.0010997.s003.doc]

|  | Time to recovery |  |  | Time to death |  |
| --- | --- | --- | --- | --- | --- |
|  | *n* = 135, *n_ind_* = 399 |  |  | *n* = 122, *n_ind_* = 333 |  |
| Parametric model | AIC | K |  | AIC | K |
| Exponential | 1188.7 | 3 |  | 503.0 | 2 |
| Gaussian | 1201.3 | 4 |  | 556.2 | 3 |
| Logistic | 1201.8 | 4 |  | 559.2 | 3 |
| Loglogistic | 1108.6* | 4 |  | 461.8* | 3 |
| Weibull | 1125.6 | 4 |  | 478.9 | 3 |

*n, n_ind_*, total number of experiments and individuals, respectively, in each data set

* The *P* values for the Grønnesby and Borgan statistics (Goodness-of-fit test) indicated good calibration for the selected model (i.e. loglogistic model): *P*≥0.11 for the time to recovery data and *P*≥0.52 for the time to mortality data
