# Supplementary material for: Model-Based Evaluation of Highly and Low Pathogenic Avian Influenza Dynamics in Wild Birds
Source: PLoS One. 2010 Jun 23;5(6):e10997. doi: 10.1371/journal.pone.0010997 (PMC2890401; doi:10.1371/journal.pone.0010997)
Supplement: Table S2 — Sensitivity of epidemiological parameters for peak prevalence and proportion of the population dying from AI. The model describes disease dynamics in a population of 10000 individuals during 60 days. We used Latin Hypercube Sampling (N = 30, 100 runs) and a semi partial correlation coefficient (SPC) to measure the relative influence of model parameters. See text for additional information. (0.09 MB DOC) [file pone.0010997.s004.doc]

|  |  | Peak prevalence |  |  |  | Population mortality |  |
| --- | --- | --- | --- | --- | --- | --- | --- |
| Parameter | Distribution ^a^ | LP |  | HP |  | HP |  |
|  |  | Adult | Young | Adult | Young | Adult | Young |
| Rate of infectious contact θ | *U*(0, 1) | 0.39±0.09 ^c^ | 0.37±0.11 | 0.76±0.07 | 0.64±0.08 | 0.04±0.04 | 0.05±0.08 |
|  |  | <0.001 ^d^ | <0.001 | <0.001 | <0.001 | 0.694 | 0.609 |
| Infection rate τ | *N*(mean, SD^2^) ^b^ | 0.37±0.10 ^e^ | 0.40±0.09 ^e^ | 0.18±0.09 ^e^ | 0.40±0.10 | 0.01±0.04 | 0.10±0.13 |
|  |  | <0.001 | <0.001 | 0.077 | <0.001 | 0.931 | 0.327 |
| Transition rate σ (E→I) | *N*(mean, SD^2^) | 0.20±0.15 | 0.19±0.14 ^e^ | 0.00±0.07 | 0.06±0.10 | 0.00±0.04 | -0.02±0.07 |
|  |  | 0.050 | 0.058 | 0.965 | 0.413 | 0.963 | 0.864 |
| Scale of recovery rate γ * | *N*(mean, SD^2^) | 0.21±0.15 | 0.14±0.14 | 0.32±0.08 | 0.17±0.10 | 0.88±0.07 | 0.60±0.10 |
|  |  | 0.038 | 0.168 | 0.001 | 0.096 | <0.001 | <0.001 |
| Scale of mortality rate *d* * | *N*(mean, SD^2^) | n/a | n/a | -0.01±0.08 | 0.19±0.11 | -0.25±0.04 | -0.63±0.11 |
|  |  | n/a | n/a | 0.915 | 0.056 | 0.013 | <0.001 |

n/a, not applicable

^a^ Parameter values for the distribution were determined from rate and SD provided in Table 1.

^b^ Mean semi partial correlation coefficient (SPC) ± SD.

^c^ z-test probability of rejecting the null hypothesis that the mean SPC does not differ from 0.

^d^ In the case of LPAI infection, we used a uniform distribution *U*(0, 10) for τ because of the large SD (τ = 2.44±3.96).

^e^ SPC are based on both adult and young bird combined

* In the loglogistic model, increasing the scale parameter leads to a decrease of the hazard rate
